# Potential Roles of Long Noncoding RNAs as Therapeutic Targets in Renal Fibrosis

**DOI:** 10.3390/ijms21082698

**Published:** 2020-04-13

**Authors:** Hyun Jin Jung, Hyun-Ju Kim, Kwan-Kyu Park

**Affiliations:** 1 Department of Urology, College of Medicine, Catholic University of Daegu, Gyeongsan 42472, Korea; hnjini@cu.ac.kr; 2 Department of Pathology, College of Medicine, Catholic University of Daegu, Gyeongsan 42472, Korea; bale4450@naver.com

**Keywords:** long noncoding RNA, renal fibrosis, therapeutic target

## Abstract

Many studies have made clear that most of the genome is transcribed into noncoding RNAs, including microRNAs (miRNAs) and long noncoding RNAs (lncRNAs), both of which can affect different cell features. LncRNAs are long heterogeneous RNAs that regulate gene expression and a variety of signaling pathways involved in cellular homeostasis and development. Several studies have demonstrated that lncRNA is an important class of regulatory molecule that can be targeted to change cellular physiology and function. The expression or dysfunction of lncRNAs is closely related to various hereditary, autoimmune, and metabolic diseases, and tumors. Specifically, recent work has shown that lncRNAs have an important role in kidney pathogenesis. The effective roles of lncRNAs have been recognized in renal ischemia, injury, inflammation, fibrosis, glomerular diseases, renal transplantation, and renal-cell carcinoma. The present review focuses on the emerging role and function of lncRNAs in the pathogenesis of kidney inflammation and fibrosis as novel essential regulators. Although lncRNAs are important players in the initiation and progression of many pathological processes, their role in renal fibrosis remains unclear. This review summarizes the current understanding of lncRNAs in the pathogenesis of kidney fibrosis and elucidates the potential role of these novel regulatory molecules as therapeutic targets for the clinical treatment of kidney inflammation and fibrosis.

## 1. Introduction

Data analyses from genome projects have shown that less than 2% of transcribed genes encode protein-coding RNA, and that most noncoding transcripts are more than 200 base pairs and make up a group of long noncoding RNA (lncRNA) [1,2,3]. These lncRNAs may have their own promoters and be located between protein-coding genes [4]. Emerging evidence shows that lncRNAs play key roles in a variety of biological processes such as proliferation and/or apoptosis through complicated mechanisms, and are also important in the development of kidney diseases [5]. Recently, lncRNAs were found to have potential use in diagnosis, and as biological markers in predicting prognosis and targets for the treatment of incurable diseases [6], particularly as highly disease-specific ideal targets in kidney disease [7,8]. Although lncRNAs are important players in the initiation and progression of many diseases, the role of those associated with renal fibrosis remains largely undefined [9].

LncRNAs are tissue-specific, and their expressions in human organs is lower than protein-coding genes [10]. Compared to protein-coding RNAs, lncRNAs are highly specific to organs and cell types, making them candidates as diagnostic biomarkers and gene-therapy targets [11]. Although lncRNAs are found in both the nucleus and cytoplasm, many of them are predominantly found in the nucleus, suggesting their important role in gene transcription [12,13,14]. Many studies have demonstrated that lncRNA is an important regulatory molecule that can be targeted to modulate cellular physiology and functions in the pathophysiological progress of tumors, and of autoimmune and cardiovascular diseases [12,15,16,17,18,19]. The functional roles of lncRNAs in kidney pathogenesis have been unfolded in the past few years [20,21].

The effective roles of lncRNAs in kidney disease have been recognized in renal ischemia, injury, inflammation, fibrosis, glomerular diseases, renal transplantation, and renal-cell carcinoma [22]. Huang et al. [23] reported that lncRNA, activated by TGF-β1, is a promising biomarker in predicting the acute rejection (AR) of renal allografts. However, their underlying mechanisms are still poorly understood. Recent application of next-generation sequencing has increased our knowledge of lncRNAs relating to various chronic fibrosis-related diseases [24]. Zhou et al. [25,26] identified a number of Smad3-associated lncRNAs that are related to renal inflammation and fibrosis in mouse models of kidney disease, suggesting that TGF-β/Smad3-associated lncRNAs may have regulatory roles in renal fibrosis and inflammation. However, little is known about how lncRNAs regulate TGF-β1/Smad signaling in kidney disease. Then, a total of 17 lncRNAs were selected after searching the PubMed for the key words of kidney, fibrosis, and lncRNA (Table 1). 

The present review summarizes the latest advances in understanding the regulatory functions of lncRNAs, and their roles in the prevention and processes of renal fibrosis. Understanding the molecular mechanism of lncRNAs in pathophysiological kidney processes may contribute to developing more effective therapeutics to prevent renal fibrosis.

## 2. Classification and Mechanisms of lncRNA Functions

NcRNAs are classified according to size as small (shorter than 200 nucleotides) and long (longer than 200 nucleotides) transcripts. Small ncRNAs, including miRNAs and small interfering RNAs (siRNA) are involved in the regulation of various biological processes in kidney diseases [27]. Many lncRNAs are not easily classified across particular categories, and the same lncRNAs may be listed in different groups in all classifications [5,28]. Moreover, since the vast majority of lncRNAs remain functionally uncharacterized, the classification is difficult.

It was reported that lncRNAs can regulate the expression of target genes via a variety of mechanisms, although complete understanding is yet to be achieved [29,30,31,32]. Many studies demonstrated that these molecules play critical roles in the regulation of specific cellular processes, specifically in protein-coding gene expression at the epigenetic, transcriptional, and post-transcriptional levels [29,33,34,35,36]. Taken together, these distinct molecular mechanisms allow dysregulated lncRNAs to up- or downregulate gene expression, thereby demonstrating their regulatory functions in various biological processes. The complicated mechanisms that underlie such regulatory behaviors require further investigation.

**Figure 1:**
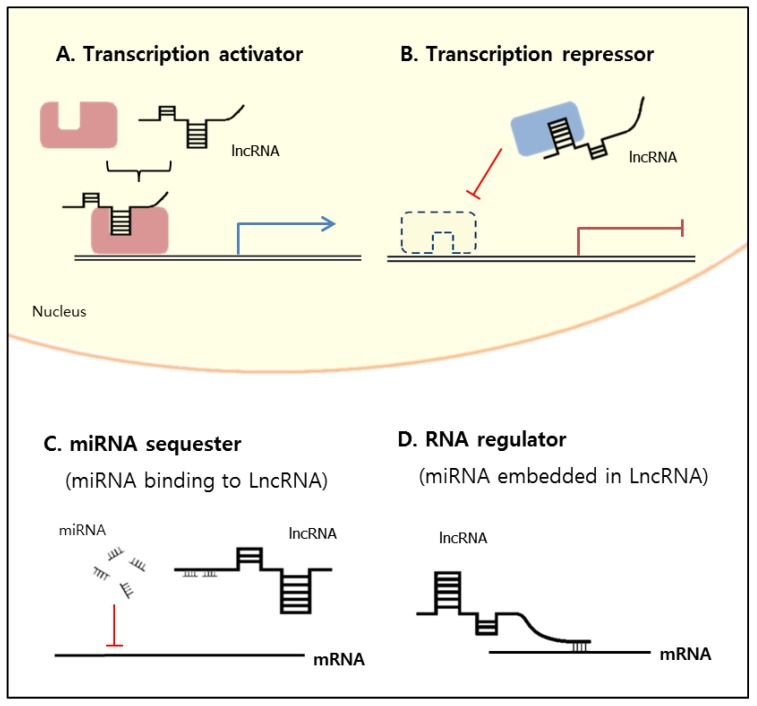
Long noncoding RNA (lncRNA) functions at nucleus and cytoplasm. (**A**) Transcription activator involved in gene expression and transcription regulation in nucleus; (**B**) transcription repressor inhibits gene expression by occupying DNA binding site in nucleus; (**C**) miRNA sequester can function as miRNA decoy to sequester miRNAs from their mRNA targets; (**D**) RNA regulator decreases mRNA expression as RNA regulator in cytoplasm.

Here, on the basis of previous reports [37], we suggest that the presumed molecular mechanisms of lncRNAs are divided into four categories according to their function at the nucleus or cytoplasm (Figure 1): (1) transcription activator, binding to the transcription factor, controls the epigenetic state of particular genes, and is involved in transcriptional regulation in the nucleus (Figure 1A); (2) transcription repressor, also binding to the transcription factor, inhibits gene expression by occupying the DNA-binding site of the transcription factor in the nucleus (Figure 1B); (3) miRNA sequester, binding with miRNAs, causes increased translation efficiency of mRNA in the cytoplasm (Figure 1C); and (4) RNA regulator competes with miRNA-mediated inhibition by, for example, having miRNA embedded in the lncRNA, leading to decreased mRNA expression in the cytoplasm (Figure 1D). There has been, however, no systematic and unambiguous classification of lncRNAs to date, and many existing classifications are conflicting and overlapping [24].

For their epigenetic role, the majority of lncRNAs localize to the nucleus, and increasing evidence indicates that nuclear lncRNAs epigenetically regulate gene expression by altering the activity of the transcription factor [38]. At the transcriptional level, lncRNAs regulate gene expression by guiding transcription factors to the promoter region of target genes to regulate their transcription, and by functioning as transcriptional activators or repressors to mediate gene transcription [5,39,40,41,42,43,44]. At the post-transcriptional level, lncRNAs regulate the expression of genes responsible for biological functions by modulating mRNA stability, translation, and degradation [24].

Unlike miRNAs, which are RNA segments of 22 nucleotides in length and function exclusively at the post-transcriptional level [45], lncRNAs participate in both the transcriptional and post-transcriptional regulation of genes. LncRNAs can act as sponges for miRNAs and thereby negatively regulate them [46,47]. Another regulatory mechanism through which lncRNAs control gene expression is by controlling miRNA biogenesis, distribution, and degradation [48]. Although the underlying mechanisms are still largely unknown, the tissue- and disease-specific characteristics of lncRNAs make them a potential biomarker and therapeutic target for clinical settings.

## 3. LncRNAs Related to Renal Fibrosis

As discussed, lncRNAs are involved in renal inflammation and fibrosis mediated by TGF-β1/Smad3 [26]. By using RNA sequencing, Zhou et al. [26] identified a number of Smad3-dependent lncRNAs related to renal fibrogenesis in mice with kidney injury induced by unilateral ureteral obstruction (UUO), and in anti-glomerular basement membrane glomerulonephritis models. Elsewhere, Lorenzen et al. [49] presented lncRNA as a novel prognostic AR biomarker. These validated features represent new potential clinical applications of lncRNAs to monitor rejection episodes without employing invasive allograft biopsies. Chen et al. [50] investigated the expression profiles of lncRNAs in kidney-biopsy samples of recipients with AR using a microarray technique, exploring the correlation between 32 lncRNAs and 12 miRNAs in controlling regulatory networks in AR development [51], demonstrating that lncRNAs are possibly involved [50]. Later, differential expression of lncRNAs was detected in the allograft biopsy and urine samples of recipients with acute T-cell-mediated rejection [49].

Recent studies have demonstrated that lncRNAs are functionally important in the development of diabetic nephropathy (DN) in response to risk factors that also regulate key inflammatory factors, such as NF-κB and others [48,52]. In particular, Sun et al. [53] examined the pathogenic role of this novel Smad3-dependent lncRNA in Type 2 diabetic nephropathy (T2DN). In addition, Wang et al. [54] utilized high-throughput technology to identify abnormally expressed lncRNAs and nearby mRNAs in a mouse DN model, and to determine the role of candidate lncRNAs in the proliferation and fibrosis of mesangial cells as prominent features of early-stage DN.

### 3.1. Arid2-IR

Zhou et al. [25] characterized the lncRNA np_28496 and, finding it located within the intron region of the Arid2 gene, named it Arid2-IR. The functional role of the molecule and its therapeutic potential for targeting in renal fibrosis and inflammation were investigated, and it was found that the promoter region of Arid2-IR contained a Smad3-binding site, and deletion of the Smad3 gene blocked the upregulation of Arid2-IR in UUO kidneys, suggesting a positive regulatory role for Smad3 in Arid2-IR expression during renal inflammation. The differential effects of Arid2-IR in renal fibrosis and inflammation suggest that Arid2-IR may be a downstream mediator of Smad3 in renal inflammation because mice null for Smad3 are protected against Smad3-mediated fibrosis and NF-κB-driven inflammation in a number of renal diseases [55,56,57]. Further investigation revealed that Arid2-IR stimulates the NF-κB-dependent renal inflammatory pathways without effect on TGF-β1/Smad3-mediated renal fibrosis in vitro and in vivo [25]. Additionally, the treatment of an obstructed kidney with short hairpin lncRNA-Arid2-IR (shRNA) blunted NF-κB-driven renal inflammation without any effect on TGF-β1/Smad3-mediated renal fibrosis [25]. Thus, Arid2-IR is a lncRNA that promotes NF-κB-dependent renal inflammation, and blocking it may therefore represent a novel and specific therapy for inflammatory kidney disease. However, the precise molecular mechanisms between Arid2-IR and NF-κB pathway-related signaling proteins require further investigation.

### 3.2. CHCHD4P4 (+EMT)

LncRNAs can play a regulatory role in the development of epithelial-to-mesenchymal transition (EMT), but it is not known whether they are involved with or influence EMT in renal tubular epithelial cells. Zhang et al. [58] demonstrated that lncRNA CHCHD4P4 promotes EMT and inhibits cell proliferation in injured renal proximal tubular epithelial cells, suggesting that CHCHD4P4 plays a critical role in the process of renal fibrosis. They explored the roles of lncRNAs in glyoxylate-exposed and healthy mouse kidneys using microarray technology. A total 376 mouse lncRNAs were differentially expressed between the two groups. Fifteen lncRNA homologs, including AU015836 and CHCHD4P4, were identified in mice and humans. Further research is necessary to explore the mechanism by which EMT-related genes are regulated by CHCHD4P4 in kidney diseases.

### 3.3. CYP4B1-PS1-001

LncRNA CYP4B1-PS1-001 regulates the proliferation and fibrosis of mesangial cells in DN. Wang et al. [59] found that the role of CYP4B1-PS1-001 in the proliferation and fibrosis of mesangial cells as prominent features during early-stage DN could provide a potential therapeutic target and molecular biomarker for the disease. LncRNA microarrays were used to detect lncRNAs in three cases of kidney tissue from db/db mice with DN. In that study, CYP4B1-PS1-001 was significantly downregulated in response to DN in vitro and in vivo, while its overexpression inhibited mesangial proliferation and fibrosis in diabetic conditions [59].

### 3.4. ENSMUST00000147869

Microarray analysis was used to screen for abnormally expressed lncRNAs and nearby mRNAs in the renal tissue of DN and normal mice [54]. Overexpression of ENSMUST00000147869 was seen to significantly reduce the proliferation and fibrosis indices of, for example, PCNA and cyclin D1 and, collagen I and fibronectin, respectively, and to protect mesangial cells against high-glucose-induced proliferation and injury. These results suggested the potential role of ENSMUST00000147869 in the proliferation and fibrosis of mesangial cells, providing data for its use as a molecular biomarker and therapeutic target for DN [54].

### 3.5. Erbb4-IR

Feng et al. [60] investigated another lncRNA, np_5318, which is largely upregulated in UUO kidneys and was identified as a direct Smad3 target gene [26]. Np_5318 was characterized as 2310-nt noncoding RNA located within the intron region of Erbb4 on chromosome 1 of the mouse genome, and was named Erbb4-IR. Erbb4-IR is a novel lncRNA responsible for TGF-β1/Smad3-mediated renal fibrosis and it is a specific therapeutic target for chronic kidney disease (CKD) [48,60]. Silencing Erbb4-IR was shown in vitro to block TGF-β1-induced collagen I and α-SMA expressions, and effectively attenuate renal fibrosis by inhibiting TGF-β1/Smad3 signaling [60]. Erbb4-IR was highly upregulated in the kidneys of mice with UUO, but was largely suppressed in mice lacking Smad3. As such, TGF-β1/Smad3 signaling may induce Erbb4-IR to further increase its fibrotic response by inhibiting the Smad7-dependent negative feedback loop, and the kidney-specific silencing of Erbb4-IR accordingly upregulated renal Smad 7, thus blocking TGF-β1/Smad3-mediated renal fibrosis in vivo and in vitro. This study also identified Erbb4-IR as responsible for TGF-β/Smad3-mediated renal fibrosis by downregulating Smad7. Targeting Erbb4-IR could therefore be a specific and effective therapy for CKD associated with progressive renal fibrosis [60].

Elsewhere, Sun et al. [53] found that Erbb4-IR was highly expressed in the T2DN of db/db mice, specifically induced by advanced glycosylation end products via a Smad3-dependent mechanism. It was found that the kidney-specific targeting of Erbb4-IR protected db/db mice in vivo and in vitro from the development of diabetic kidney injury through a mechanism associated with increasing miR-29b expression the Erbb4-IR–miR-29b axis, which was found to be a key mechanism of T2DN.

### 3.6. H19

H19 is a 3 kb non-coding RNA that plays an important role in renal development [9,61,62]. Xie et al. [9] demonstrated a significant upregulation of H19 expression in the TGF-β-induced fibrosis of human proximal tubular epithelial cells and UUO-induced renal fibrosis, and that the knockdown of H19 significantly attenuates renal fibrosis in vitro and in vivo. They showed that lncRNA-H19 expression was significantly upregulated in TGF-β2-induced HK-2 cell fibrosis and UUO-induced renal fibrosis in vivo. Increased H19 levels were found to alleviate the miR-17 repressive effect and increase fibronectin, a target gene of miR-17. Upregulated H19 expression and downregulated miR-17 were confirmed in animal models of renal fibrosis [9]. This study indicates that H19 upregulation contributes to kidney fibrosis, and that H19 inhibition may represent novel anti-fibrotic treatment in renal diseases [63].

### 3.7. HOTAIR

Homeobox (HOX) transcript antisense intergenic RNA (HOTAIR) is a lncRNA that locates to a boundary of the HOXC locus on chromosome 12q13.13 that is co-expressed with HOXC genes [24]. HOTAIR is upregulated in sepsis-induced kidney injury, and it promotes renal tubular epithelial cell apoptosis in kidney injury [64,65]. Zhou et al. [66] demonstrated that silencing lncRNA HOTAIR upregulated miR-124 to block the Notch1 pathway in UUO model, and thereby alleviating EMT and renal interstitial fibrosis (RIF), indicating HOTAIR as a potential target for RIF treatment. HOTAIR is also expressed in glomerular podocytes of both humans and mice and its expression is upregulated in experimental and human DKD [67].

### 3.8. LINC00936

Chen et al. [68] reported that LINC00963 was highly expressed in CRF rats, and FoxO3 was verified as a target gene of LINC00963. Lower LINC00963 expression attenuated interstitial fibrosis and oxidative stress in chronic renal failure (CRF) by activation of the FoxO signaling pathway, which helps us to understand the potential gene mechanisms of CRF, and may provide new prognostic markers for CRF treatment in the future. However, they did not thoroughly investigate how LINC00963 affects the activation of the FoxO signaling pathway and the potential mechanism in interstitial fibrosis CRF [68].

### 3.9. LncRNA-ATB

Qiu et al. [69] proposed that lncRNA-ATB, activated by TGF-β1, could be used as a novel diagnostic biomarker to identify recipients with AR and predict loss of kidney function [69]. In biopsy samples of recipients with AR, strongly increased levels of lncRNA-ATB were observed that influence renal-cell phenotypes and the nephrotoxicity of immunosuppressive drugs.

### 3.10. MALAT1

Metastasis-associated lung adenocarcinoma transcript 1 (MALAT1) is one of the most comprehensively studied lncRNAs in kidney diseases [11,70]. It is increased in the diabetic kidney and regulates hyperglycemia-induced renal fibrosis [19,71,72], MALAT1 can be induced by TGF-β1 [73], and it consistently promotes cell proliferation. MALAT1 is associated with the translocation of β-catenin, a mediator of kidney fibrosis, to the nuclei, and with the overexpression of serine/arginine-rich splicing factor 1 which is involved in inducing DN progression [74]. Further investigation is needed to identify the common pathogenic mechanism for MALAT1 in kidney diseases.

### 3.11. MIAT

The expression of myocardial infarction-associated transcript (MIAT) is significantly elevated in diabetic rats and patients [75], and MIAT knockout reduces the release of proinflammatory cytokines induced by diabetes. MIAT also plays a regulatory role in myofibroblast formation, through interacting with miRNA regulation, implicating that understanding their biology and their modulation may have the potential to counteract the development of renal fibrosis [76]. In vitro studies have demonstrated that the increased expression of MIAT and its potential target, nuclear factor erythroid-related factor 2 (Nrf2), in HK-2 cells incubated with high glucose improves the viability of renal tubular epithelial cells. Nrf2 is involved in protecting cells against induced oxidative stress after incubation with high glucose [77].

### 3.12. NEAT1

Nuclear-enriched abundant transcript 1 (Neat1) expression was observed to be elevated in acute kidney injury (AKI) [78,79]. NEAT1 was significantly upregulated in the sepsis-induced AKI patients. In addition, the suppression of NEAT1 alleviated lipopolysaccharide (LPS)-induced injury in rat Mesangial cells (RMCs) [78]. The RMCs were treated with LPS to induce cell injury. Then, the effects of NEAT1 suppression on the cell viability, apoptosis, cytokines expression, and oxidative stress in the LPS-stimulated RMCs were tested. The effects of the suppression of NEAT1 on LPS-induced cell injury were caused by inactivating the NF-κB pathway.

Li et al. [80] investigated the potential mechanism by which NEAT1 facilitates the progression of DN. The enrichment of NEAT1 was elevated in the serum of DN patients and mouse Mesangial cells (MMCs) induced by high concentration of glucose. NEAT1 overexpression accelerated proliferation, fibrosis, and EMT and restrained apoptosis of MMCs induced by a high concentration of glucose. These findings suggest that NEAT1 could be an effective diagnostic marker and therapeutic target for AKI and DN.

### 3.13. RANTES

The regulated on activation normal T-cell-expressed and secreted (RANTES) lncRNA is produced by renal tubular epithelial cells and acts as an inflammatory mediator in acute kidney injury (AKI) following ischemic reperfusion [48]. RANTES-deficient (−/−) mice showed better renal function by reducing acute tubular necrosis, serum creatinine levels, infiltration of inflammatory cells, and cytokine expressions compared to wild-type mice [81]. It was reported that hypoxia-inducible factor-1α (HIF-1α) may play a role in the production of RANTES in AKI [81].

### 3.14. PVT1

Plasmacytoma variant translocation 1 (PVT1) is the first lncRNA to be associated with end-stage renal disease in Type 1 and 2 diabetes [82,83], and its function has been characterized in diabetic kidney disease [84]. Alvarez et al. [48] knocked down PVT1 expression in mesangial cells, and analyzed RNA and protein levels. PVT1 was upregulated in high-glucose-treated mesangial cells, and proteins such as fibronectin 1, TGF-β1, plasminogen activator inhibitor 1, and collagen Type IV α1, which are involved in renal fibrosis and ECM accumulation, were also increased. These results suggested the significant pathological role of PVT1 in TGF-β1-mediated DN [84].

### 3.15. TapSAKI

It was reported that some lncRNAs, including TrAnscript Predicting Survival in AKI (TapSAKI), psoriasis-associated RNA induced by stress, are involved in the pathogenesis of AKI, and have potential as diagnostic biomarkers [85,86]. TapSAKI was identified from kidney biopsies and plasma samples from patients with AKI [11]. The plasma levels of circulating TapSAKI were correlated with disease severity and were specifically upregulated in tubular epithelial cells under hypoxia, suggesting that upregulated plasma TapSAKI can serve as an AKI prognosis predictor [87].

### 3.16. Tug1

Taurine upregulated gene 1 (Tug1) was identified as promoting diabetic kidney disease in mitochondria- and endoplasmic-reticulum-dependent mechanisms, respectively [88,89]. In mouse podocytes, Tug1 was found to regulate the expression of PGC-1α, which is involved in energy homeostasis and mitochondrial biogenesis, in that its overexpression improved mitochondrial bioenergetics and DN-related features [90]. The overexpression of TUG1 could suppress the proliferation and ECM accumulation of mesangial cells via inhibiting the PI3K/AKT pathway [91].

### 3.17. Xist

Using high-throughput RNA-sequencing, Zhou et al. [26] identified numerous lncRNAs that were differentially expressed in mouse models of CKD. In particular, they found that inactive X-specific transcript (Xist) was significantly upregulated in both tubular and glomerular cells in membranous nephropathic mice and in urine samples from patients with different types of glomerular disease [23]. These results suggested that Xist may be a potential urinary biomarker of membranous nephropathy.

## 4. Conclusions

Recent evidence indicated that lncRNAs represent a heterogeneous class of transcripts that are incompletely understood but rapidly emerging as potential drug targets and therapeutic candidates. Continued investigations based on the knowledge presented here will undoubtedly overcome the remaining challenges in the field of fibrosis-related diseases, and this review represents a potential research strategy for renal fibrosis-related lncRNAs. First, with the application of newly developed technologies such as next-generation sequencing, more new lncRNAs related with renal interstitial fibrosis could be found in the near future. Second, another new biological marker that can easily be detected in urine samples could be developed. Third, novel synthetic oligodeoxynucleotides, which are another ncRNA type that could prevent the function of fibrosis-related lncRNA, could be developed and used for clinical trials. Further studies are required to examine the toxicity and the pharmacokinetics of lncRNAs, and evaluating their biological properties is also important.

## Figures and Tables

**Table 1:** LncRNAs related to renal fibrosis.

LncRNA	Dysregulation	Related Disease/Experiment	Biological Role	Signal/Target Cell
Arid2-IR	Upregulation	UUO kidney	TGF-β/Smad3-associated renal fibrosis	NF-κB-dependent inflammation
CHCHD4P4	Upregulation	Renal damage (fibrosis)	Epithelial-to mesenchymal transition	Tubular epithelial cell
CYP4B1-PS1-001	Upregulation	Diabetic nephropathy	Regulation of fibrosis	Mesangial cell
ENSMUST00000147869	Upregulation	Diabetic nephropathy	Decreased PCNA and cyclin D1	Mesangial cell
Erbb4-IR	Upregulation	UUO kidney, diabetic nephropathy	Downregulation of Smad7	TGF-β1/Smad3 signaling
H19	Upregulation	UUO kidney	Downregulation of miR-17 expression	Tubular epithelial cell
HOTAIR	Upregulation	Acute kidney injury	Promotion of apoptosis	Notch1 pathway
LINC00936	Downregulation	Chronic renal failure	Regulation of fibrosis	FoxO signaling
LncRNA-ATB	Upregulation	Acute rejection	Loss of kidney function	Activated by TGF-β1
MALAT1	Upregulation	Diabetic nephropathy	Translocation of β-catenin; SRSF1 overexpression	TGF-β1-mediated fibrosis
MIAT lncRNA	Upregulation	Diabetic nephropathy	Nrf2 regulation	Tubular epithelial cell
NEAT1	Upregulation	Acute kidney disease, diabetic nephropathy	Proliferation of Mesangial cell	NF-κB pathway
PVT1	Upregulation	Diabetic nephropathy	ECM accumulation	TGF-β1-mediated fibrosis
Rantes lncRNA	Upregulation	Acute kidney injury, ischemic reperfusion	Produced by HIF-1α	Tubular epithelial cell
TapSAKI	Upregulation	Acute kidney injury, hypoxia	Predictor of prognosis	Tubular epithelial cell
Tug1	Upregulation	Diabetic nephropathy	Mitochondria-dependent mechanism	Podocyte
Xist	Upregulation	Glomerular disease	Urinary biomarker	Tubular epithelial and glomerular cell
